# An Unusual Association in an Uncommon Disease: Two Cases of Spontaneous Pneumomediastinum Associated with Pneumorrhachis

**DOI:** 10.1155/2016/5092157

**Published:** 2016-04-26

**Authors:** Luís Martins, Patrícia Dionísio, Susana Moreira, Alda Manique, Isabel Correia, Cristina Bárbara

**Affiliations:** Centro Hospitalar Lisboa Norte (CHLN), 1649-035 Lisboa, Portugal

## Abstract

Pneumomediastinum, the presence of free air in the mediastinum, is described as spontaneous pneumomediastinum when there is no apparent cause such as trauma, surgery, interventional procedures, or intrathoracic infections. Pneumorrhachis is a rare clinical condition, consisting of intraspinal air. The main causes are iatrogenic, traumatic, and nontraumatic. Spontaneous mediastinum is usually associated with subcutaneous emphysema and, occasionally, with pneumothorax; however, its association with pneumorrhachis is extremely rare. Here, we present two rare cases of spontaneous pneumomediastinum associated with pneumorrhachis caused by vigorous coughing.

## 1. Introduction

Free air can reach the mediastinum from intrathoracic (esophagus, trachea, bronchial tree, lung, and pleural space) or extrathoracic (head, neck, and peritoneum) structures [1]. It is classified as spontaneous pneumomediastinum (SPM) when there is no apparent cause such as trauma, surgery, procedures, or intrathoracic infection [2, 3]. SPM is uncommon but probably accounts for underdiagnosis.

Pneumorrhachis (PR) is a clinical entity, consisting of intraspinal air. Its etiology is usually traumatic or iatrogenic, following invasive procedures [4]. The nontraumatic or spontaneous pneumorrhachis is very rare but can occur as a complication of SPM [5].

We present two cases of SPM with pneumorrhachis caused by vigorous coughing. Despite the potential for serious complications, both cases had a successful clinical outcome. To our knowledge, there are very few reported cases of SPM associated with pneumorrhachis.

## 2. Clinical Case

### 2.1. Case  1

A 22-year-old healthy young male, who was never a smoker, presented with chest pain after an acute bout of coughing. He had a five-day history of dry cough. There was no history of trauma, fever, vomiting, or previous respiratory diseases such as asthma. On examination, he was hemodynamically stable; cervical subcutaneous emphysema was identified with crepitus on his neck and both shoulders.

**Figure 1:**
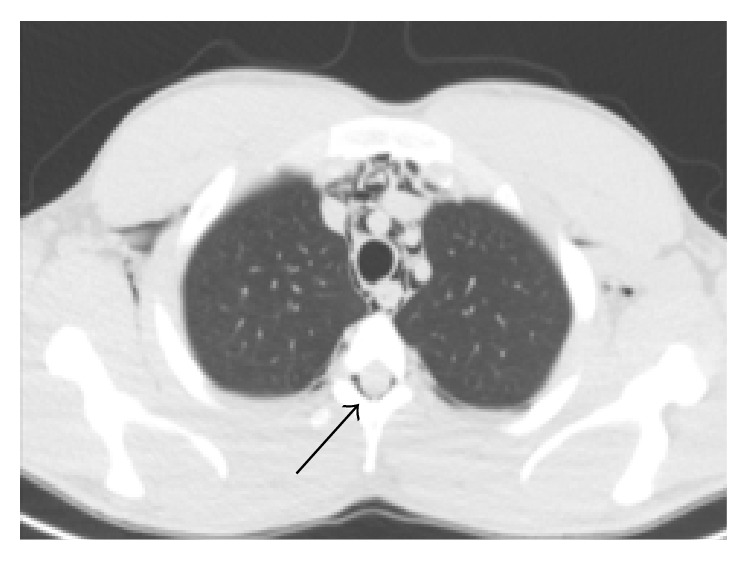
CT lung windows showing pneumomediastinum and pneumorrhachis (black arrow).

Upon percussion and auscultation, the patient's breath sounds were clear and bilaterally equal. The chest radiograph confirmed subcutaneous emphysema over the neck and chest but no pneumothorax was identified. A CT scan of the neck, chest, and abdomen was performed revealing a pneumomediastinum as well as air in the spinal epidural space at cervical and thoracic levels (Figure 1). Again, no pneumothorax was identified. The patient was admitted for observation and treated conservatively with bed rest, oxygen, analgesia, and weak opiates for cough control. Over the next four days, he made a complete recovery.

### 2.2. Case  2

**Figure 2:**
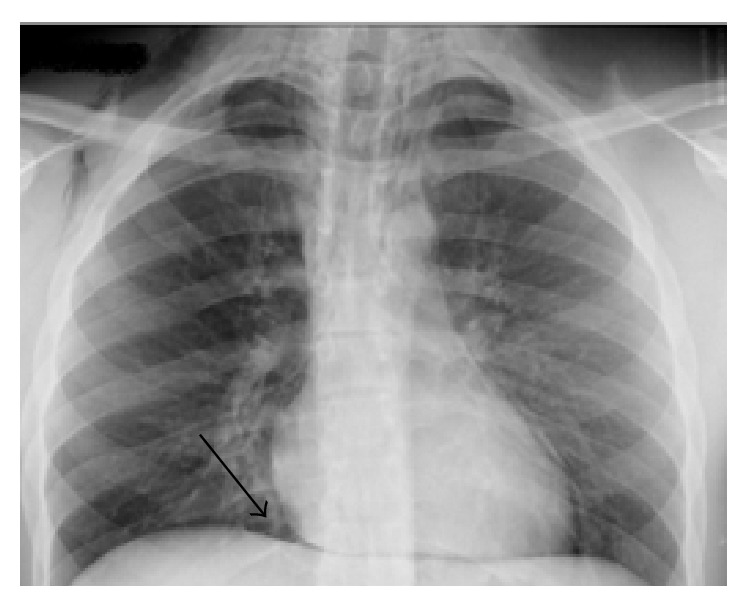
Posteroanterior chest radiograph showing subcutaneous emphysema and the continuous diaphragm sign (black arrow).

A healthy 20-year-old male who was never a smoker and with no history of trauma, drug abuse, or respiratory disease presented with retrosternal pain and dyspnea, preceded by a three-day history of irritative cough and odynophagia. On physical examination, he was found to have bilateral cervical subcutaneous emphysema. The chest radiography revealed the continuous diaphragm sign [6] (Figure 2) and a chest CT confirmed a pneumomediastinum with further dissection of air into the neck and the spinal epidural space. The patient was admitted for observation and responded well to conservative treatment with bed rest, oxygen, and opiates for cough control. All symptoms resolved within five days.

## 3. Discussion

SPM is a rare condition, first described by Hamman [7]. It is defined as the presence of interstitial air in the mediastinum that occurs spontaneously, hence not associated with surgery, trauma, organ rupture, mechanical ventilation, or intrathoracic infection [8]. The incidence of SPM has not been clearly established since data consists of reports that are case studies or small case series [3]. The incidence appears to vary depending on the case series. In a ten-year retrospective study of patients admitted to the emergency department of a university hospital, the incidence of patients diagnosed with spontaneous pneumomediastinum was 1 : 113,000 [9]. The condition primarily affects males in their second to fourth decades of life. The most common predisposing factor is a past medical history of lung disease [10]. According to a systematic review of SPM, 22% of patients had lung comorbidities, asthma being the most frequently associated condition [9]. The precipitating factors are those that increase intrathoracic pressure (emesis, cough, asthma exacerbation, defecation, physical exertion, labour, upper airway infection, neonate respiratory distress syndrome, and inhaled drug use). Moreover, spirometry and seizures have been reported to trigger SPM [11]. In some cases, a precipitating factor is not identified.

The mechanism for developing SPM is based on the Macklin effect, consisting of increased intra-alveolar pressure due to an increase in intrathoracic pressure, causing the rupture of the peripheral pulmonary alveoli. The air diffuses to the interstitial space, through the peribronchial and perivascular fascial sheath to the hilum and the mediastinal soft tissue layers. This happens because the mediastinum has a lower pressure than the lung periphery [12].

The most common symptoms of SPM are chest pain, dyspnea, cough, neck pain, and dysphagia [10]. Chest pain was the main complaint reported by our patients. On clinical examination, the most common finding was subcutaneous emphysema.

According to Sahni et al. [10], in a review of clinical series, subcutaneous emphysema was found in 58% of patients, followed by Hamman's sign in only 18% of the patients. Hamman's sign is pathognomonic of pneumomediastinum and is described as a crunching or bubbling sound over the mediastinum, synchronous with heartbeat. In our patients, the only physical finding was subcutaneous emphysema. Chest radiography may not always be enough for diagnosis, although various radiographic signs have been described [1, 6]. As reported by Kaneki et al. [13], up to 30% of patients with SPM have normal chest radiographs. A thoracic CT is considered the gold standard for the diagnosis of pneumomediastinum as it can detect mediastinal air that cannot be seen on a chest radiograph [14] and simultaneously exclude lung pathology. In our cases, the chest radiographs were suggestive of SPM, especially in the second case, where the continuous diaphragm sign was identified. This happens because of air interposition between the diaphragm and the heart, allowing for the visualization of the entire diaphragm from one side to the other [6]. The investigation of our patients did not show any underlying pulmonary disease.

In our patients, the trigger seems to be the cough-related Valsalva maneuver. Performing endoscopy, bronchoscopy, and/or contrast-swallow study can be useful; however, some authors do not recommend their routine use, unless severe symptoms or inflammatory signs are present [14, 15]. None of these were found in our patients. The most serious differential diagnosis of SPM is Boerhaave's syndrome which should be ruled out with a contrast-swallow study when suspected, especially if there is a history of forceful vomiting, because it may lead to mediastinitis [16].

The treatment of SPM is mostly conservative and consists of bed rest, oxygen therapy, and treating underlying causes [2, 3, 8]. SPM has a benign clinical course and complications are rare. Still, patients should be admitted for close monitoring of potential life-threatening complications such as air leaking into the pericardial sac, causing a pneumopericardium [17, 18]. Also, air flow through pleural structures can cause pneumothorax or even tension pneumothorax, requiring a chest tube [12, 15].

One of the potential and rare complications associated with SPM is pneumorrhachis, which was found in our patients. Pneumorrhachis results from the communication between the posterior mediastinum and epidural space since there are no fascial barriers to prevent it [19]. Air typically collects in the posterior epidural space because of its lower resistance compared with the anterior vascular network [20]. Pneumorrhachis occurs in a variety of settings, including skull and spinal fractures, epidural abscess, epidural anesthesia, lumbar puncture, and traumatic pneumothorax. Pneumorrhachis in the context of trauma indicates severe injury [21].

Our patients presented with asymptomatic spontaneous pneumorrhachis associated with SPM, which was successfully managed conservatively. Neurological symptoms and signs due to the pressure effect of pneumorrhachis are rarely observed. However, several cases of symptomatic nontraumatic pneumorrhachis causing cervical myelopathy [22], radicular pain, and paraplegia [23], as well as mild headache [24], are described. The follow-up of patients with SPM is not well defined. The majority of studies concluded that long-term follow-up is usually unnecessary because recurrence is rare, although there are some cases of recurrent SPM published in the literature [3, 8].

In conclusion, SPM is an underdiagnosed condition and a high index of suspicion is needed, especially in young patients presenting with chest pain and/or dyspnea. In line with the two clinical cases described and in accordance with the literature, conservative management is recommended. SPM is usually asymptomatic and self-remittent, with or without pneumorrhachis [19]; however, prompt diagnosis and close monitoring remain important in order to avoid potential serious complications.
